# IGF2BP2 promotes lncRNA DANCR stability mediated glycolysis and affects the progression of FLT3-ITD + acute myeloid leukemia

**DOI:** 10.1007/s10495-023-01846-0

**Published:** 2023-04-15

**Authors:** Shenghao Wu, Changwei Chi, Shanshan Weng, Wenjin Zhou, Zhen Liu

**Affiliations:** Department of Hematology, The Dingli Clinical College of Wenzhou Medical University (The Second Affiliated Hospital of Shanghai University, Wenzhou Central Hospital), Wenzhou city, Zhejiang Province China

**Keywords:** Acute myeloid leukemia (AML), FLT3-ITD, IGF2BP2, N6-methyladenosine, Glycolysis

## Abstract

Internal tandem duplication (ITD) is the most common type of FLT3 mutation (FLT3-ITD), accounting for about 25% of AML patients. The expression of DANCR in FLT3-ITD AML had not been paid attention to, and whether its regulatory relationship with IGF2BP2 can affect the progression of FLT3-ITD AML was unclear. Our study sought to verify the biological role of IGF2BP2 as an m6A reading protein in FLT3-ITD AML. To further explore the role and mechanism of DANCR in AML, and provide a basis for the screening of biomarkers and the development of targeted drugs. The results show that IGF2BP2 was upregulated in FLT3-ITD+ AML patients and cells. Si-IGF2BP2 could inhibit the proliferation, glycolytic and promote the apoptosis in MV4-11 cells. IGF2BP2 could promote the DANCR RNA stability. This discovery will provide new horizons for early screening and targeted therapy of FLT3-ITD+ AML.

**Supplementary Information:**

The online version contains supplementary material available at 10.1007/s10495-023-01846-0.

## Introduction

leukemia, commonly known as “blood cancer”, is a kind of blood malignant disease characterized by clonal proliferation of hematopoietic stem cells. Clonal leukemia cells proliferate and accumulate in bone marrow and other normal hematopoietic tissues, inhibit hematopoietic function, and infiltrate into other non-hematopoietic tissues and organs through blood circulation, resulting in organ failure and poor prognosis [1]. Clinical manifestations often include anemia, bleeding, infection and fever. Acute myeloid leukemia (AML) is a common type of leukemia, accounting for 80% of acute leukemias, and has a high incidence in children. 70% of AML patients over 65 years of age will die within one year of diagnosis, with a high mortality rate [2]. The pathogenesis of AML is complex and diverse. Chemical substances, radioactive substances, genetic factors, gene mutations, abnormal signaling pathways, epigenetic regulation, leukemia microenvironment or immune imbalance can all cause AML [3].

Fgs-like tyrosine kinase 3 (FLT3) is a type III receptor tyrosine kinase, which is composed of five immunoglobulin-like domains in the extracellular region, a paramembrane domain and a tyrosine kinase (TK) domain [4]. FLT3 plays an important role in the survival, proliferation and differentiation of hematopoietic cells. FLT3 plays an important role in the early stages of both myeloid and lymphoid lineage development and is bound and activated by FLT3 ligands. When FTL3 binds to its ligand, it dimerizes and autophosphorylates to activate tyrosine kinase activity, which in turn activates PI3K/Akt and Ras/MAPK pathways, resulting in DNA damage and repair defects, and increases cell proliferation and anti-apoptosis [5]. Clinically, FLT3 gene mutation is the most common genetic change and poor prognosis factor in AML patients. FLT3 mutation is found in most AML cells, which activates anti-apoptotic and pro-growth signals and stimulates the proliferation of AML cells. internal tandem duplication (ITD) is the most common type of FLT3 mutation (FLT3-ITD), accounting for about 25% of AML patients. Other studies have shown that FLT3-ITD can activate Rho kinase in AML cells, which leads to LIM kinase (LIMK) phosphorylation and affects cytoskeletal dynamics, cell growth and apoptosis. Other data showed that FLT3-ITD could promote serine synthesis, and the newly synthesized serine could selectively inhibit the proliferation of AML cells [3, 6]. Therefore, a deep understanding of the mechanism of action of FLT3-associated mutant AML can provide new ideas for the treatment of AML.

The morphological and functional diversity of RNA is based on extensive modifications of the four typical bases of RNA. Since the 1960s, more than 150 chemical modifications of RNA have been discovered [7]. Among them, m6A modification is the most common post-transcriptional modification in eukaryotes by adding methyl groups to the N-6 position of adenosine residues. m6A modification is dynamically controlled by methylated transferases (writers), demethylated transferases (erasers), methylation-related reading proteins (readers), and other proteins that may influence these regulation. m6A modification plays a key role in gene expression by affecting different stages of mRNA splicing, nuclear export, stability and translation [8].

Readers include YTH-domain-containing protein families, insulin-like growth factor 2 mRNA binding proteins (IGF2BPs), RNA-binding proteins and “indirect” readers. The YTH-domain-containing protein family, which includes YTHDF1, YTHDF2, YTHDF3, YTHDC1 and YTHDC2, is a group of major m6A readers, It plays an important role in ncRNA and mRNA stability, mRNA splicing, mRNA structure, mRNA export, translation efficiency and miRNA biogenesis. IGF2BPs, including IGF2BP1/2/3, are a unique class of m6A readers that improve ncRNA and mRNA stability by recognizing GGC (m6A) sequence (typical m6A sequence) for targeted binding to target RNA. In addition, “indirect” readers HNRNPC and HNRNPG can be used as structural “switches” that preferentially bind m6A modifications [9].

IGF2BP2 was involved in the methylation catalytic process as a reader. Current studies have shown that IGF2BP2 could participate in various tumor processes including AML [10, 11]. At present, studies have shown that IGF2BP2 can participate in stabilizing lncRNA and promoting its expression, thereby regulating the proliferation, invasion and metastasis of various tumors, including nasopharyngeal carcinoma and pancreatic cancer [12–16]. Differentiation antagonistic non-protein-coding RNA(DANCR) is a common lncRNA in the cytoplasm, which exists on human chromosome 4q12. At present, previous studies have shown that DANCR expression is significantly increased in a variety of tumors and has cancer-promoting effects [17, 18]. However, the expression of DANCR in FLT3-ITD AML had not been paid attention to, and whether its regulatory relationship with IGF2BP2 can affect the progression of FLT3-ITD AML was unclear. Therefore, this study sought to verify the biological role of IGF2BP2 as an m6A reading protein in FLT3-ITD AML. To further explore the role and mechanism of DANCR in AML, and provide a basis for the screening of biomarkers and the development of targeted drugs.

## Materials and methods

### Clinical sample

The samples were collected from 60 patients who were diagnosed as AML (including 30 FLT3-ITD + AML patients, 30 FLT3-ITD- AML patients) and 30 healthy donors. Among those participants, the gender ratio approached 1:1 and the ages of them were between 30 and 60. All materials were admitted to Wenzhou Central Hospital. The samples were exacted out of patients and volunteers by operating and then were stored at − 80 °C for use.

### Cell culture and transfection

Human cell line Molm-13 (FLT3-ITD + cell line), MV4-11 (FLT3-ITD + cell line), THP-1 (FLT-ITD wild type) and U937 (FLT-ITD wild type) were obtained from ATCC (Manassas, VA, USA) and was cultured in DMEM. Medium supplemented were with 10% FBS (fetal bovine serum) storing at 37 °C with 5% CO_2_.

The plasmid vectors pcDNA3.1-IGF2BP2 (oe-IGF2BP2), pcDNA3.1-DANCR (oe-DANCR), siRNA-IGF2BP2 (si-IGF2BP2), the plasmid vectors PCDNA3.1-IGF2BP2 pcDNA3.1-PKM (oe-PKM) and related negative controls were transfected into MV4-11 cells. Transfection Cells were transfected using the Lipofectamine 2000 kit according to the manufacturer’s instructions. BLOCK-iTTM RNAi Designer, an RNAi sequence design software, was used to design interference nucleotide sequences, and unrelated nucleic acid sequences with the same number of bases were designed and synthesized as negative controls. The positive clones were selected and the recombinant plasmids were extracted and sequenced. Liposome transfection, 24 h before transfection, cells in logarithmic growth phase were taken, digested by trypsin, resuspended with complete medium, and then blown and mixed with pipestraw to make cell suspension. The cells were seeded into 6-well plates at a concentration of 1 × 106 cells per well and cultured in a 5% CO_2_ incubator at 37 ℃ for 18–24 h to achieve a confluence rate of 80%~90% in each well before transfection. At 3 h before transfection, the original medium was removed and replaced with fresh basal medium without serum and antibiotics. The liposome Lipofectamine 2000 was used for transfection according to the kit instructions, and the cells were cultured for 48 h at 37 ° C with 5% CO_2_.

### RNA isolation and quantitative real-time PCR (qRT-PCR)

In this study, total RNA was isolated from tissues and cells using TRIzol reagent (Invitrogen, USA). Mirnas were extracted from tissues or cells using the miRcute miRNA Kit (Tianjian, Shanghai, China) according to the manufacturer’s instructions. Mir-X miRNA First-Strand Synthesis Kit (Taraka, China) and PrimeScript™ RT reagent Kit (Taraka, China) were used for reverse transcription of miRNA and mRNA, respectively. miScript SYBR Green PCR Kit (Qiagen, USA) and Mir-X miRNA qRT-PCR TB Green® Kit (Taraka, China) to determine the expression levels of DANCR, IGF2BP2, miR-4701-5p, GLUT1 and HK2 in tissues and cells. Follow the instructions of the kit for all operations. Quantitative real-time PCR was performed using an Applied Biosystems 7300 real-time PCR system. PCR conditions consisted of an initial holding period of 3 min at 95 °C, followed by a two-step PCR procedure consisting of 40 cycles of 94 °C for 5 s and 60 °C for 35 s. GAPDH and U6 were used as reference controls for mRNA and miRNA, respectively. All primer sequences are shown in Table S1, and all primers were designed and synthesized by Shanghai Shenggong Biological Co., LTD.

### Western blot

Protein extraction reagent (50 mM Tris HCl, pH 7.4; 120 mM NaCl; 1% Nonidet P − 40; 0.25% deoxycholate; 0.1% sodium dodecyl sulfate) were extracted and supplemented with protease inhibitor mixture (Beyotime, Shanghai, China). The protein concentrations of the samples were then normalized using the BCA Protein Assay Kit (Pierce, Rockford, IL). Protein extracts were equally added to 10% SDS polyacrylamide gels, subjected to electrophoresis, and then transferred to nitrocellulose membranes (Amersham Bioscience). After blocking with 5% nonfat milk in PBS, related primary antibodies (anti-HIF1α (ab179483, 1:1000 dilution, Abcam, Cambridge, MA, USA), anti-HK2 (ab209847, 1:1000 dilution, Abcam, USA), anti-GLUT1 (ab115730, 1:100000 dilution, Abcam, USA) and GAPDH (ab8226, 1 µg/ml, Abcam, USA)) were incubated overnight, followed by a horseradish peroxidase conjugated secondary antibody (Santa Cruz Company). The signal was detected by a chemiluminescence substrate Kit (Millipore Company, Bedford, MA, USA).

### Cell viability assay

The cell proliferation of each treatment group was determined by measuring the amount of MTT reduced to formaldehyde. MTT reagent was then supplemented, followed by cell incubation for 3 h. The solution was decanted, and 100µL DMSO was added for dissolution of purple formazan crystals. The absorbance of the resulting solution was measured at 450 nm with a microplate (Bio, USA).

### Cell apoptosis analysis

Cells were harvested from different groups for apoptosis analysis after double staining of the cells with Annexin-V and Propidiumiodide (PI) via using Annexin-V-FLOUS staining kit in the basis of the manufacturer’s protocol (Roche, Mannheim, Germany).

### TUNEL staining to detect cell apoptosis ability

Cells from each treatment group were fixed with formaldehyde for 15 min on ice and washed with PBS. Add ice-cold 70% ethanol and incubate for 30 min, pellet cells and resuspend in wash buffer and wash again. Cells were further pelleted and resuspended in staining solution and incubated at 37ºC for 60 min before adding wash buffer, pelleting cells and discarding supernatant. Cells were resuspended in propidium iodide/RNAse A solution and incubated for 30 min at room temperature before analysis by fluorescence microscopy.

### Animals

Male BALB/c nude mice (6 weeks) were provided by Slack Laboratory Animal Center (Shanghai) and maintained under specific pathogen-free conditions. A total of 12 mice were randomly divided into two groups and injected with MV4-11 cells (5 × 10^6^ cells /100 µL) of transfection vector (Ctrls) or si-IGF2BP2 into nude mice. The tumorigenesis period was controlled for 42 days and was divided into 8 tumor volume weights on average. At the end of the experiment, each individual was killed by glutaraldehyde anesthetic overdose, and the tumor was removed and weighed. The tumor volume formula was: 1/2×L^2^×W (L, length (mm); W, Tumor width (mm).

### Statistical analysis

Data were analyzed by SPSS 22.0 software and presented as mean ± standard error (SE) values of at least three independent experiments. Comparisons between the two groups were analyzed by Student-t test. One-way analysis of variance was used for comparison among multiple groups, and Tukey’s method was used for pairwise comparison after the event. In order to P&lt; 0.05 was considered statistically significant.

Other detailed methods, such as lncRNA screening, EdU staining, tissue immunofluorescence, and M6A-related gene screening, are shown in the Supplementary methods.

## Results

### The IGF2BP2 was high expressed in FLT3-ITD + AML patients and cells

**Fig. 1:**
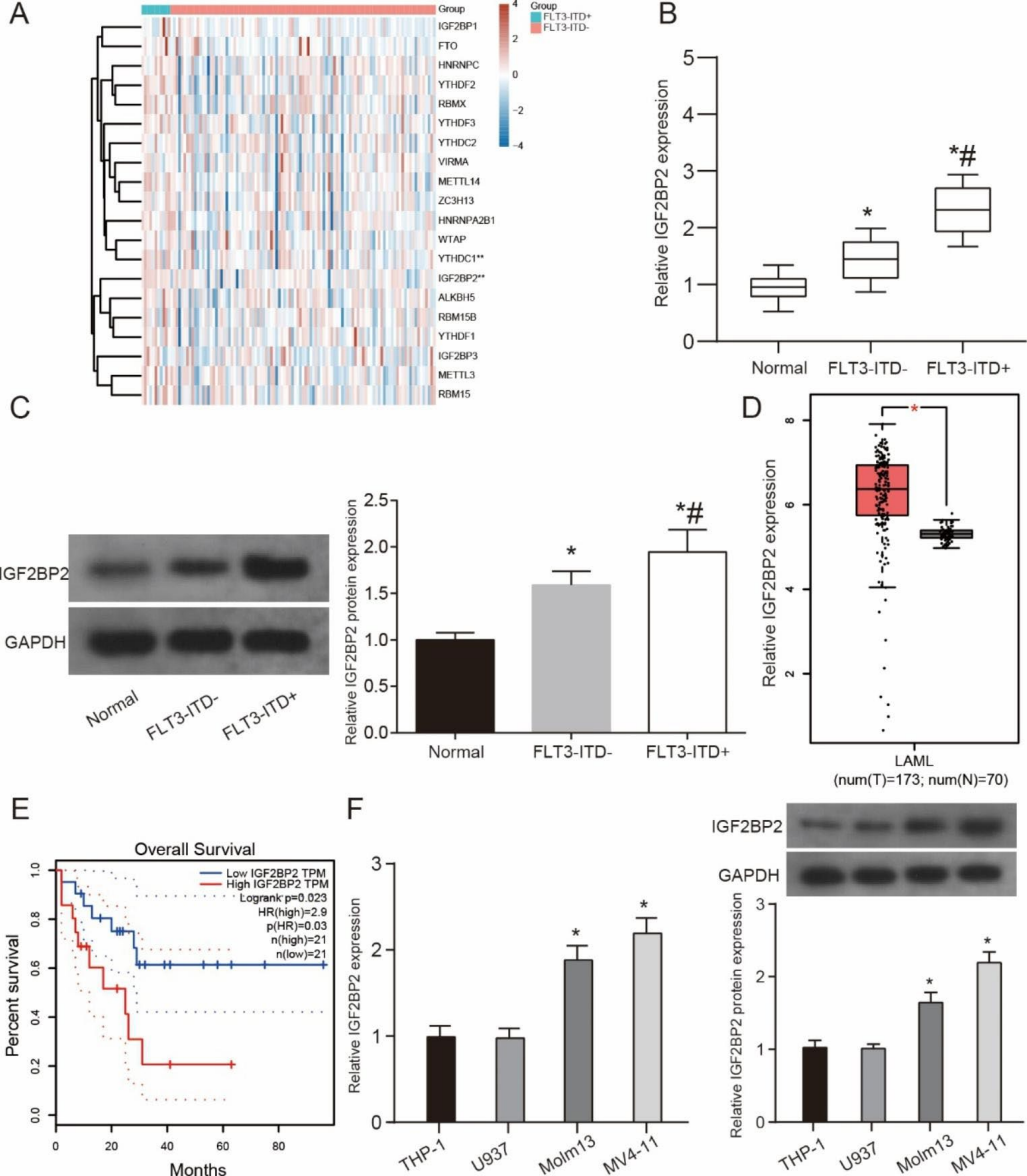
**IGF2BP2 was up-regulated in FLT3-ITD + AML** (**A**) The heat map was showed the 23 m6A-relative genes in FLT3-ITD + patients as compared to that in the FLT3-ITD- samples analyzed by TCGA database. (**B**) Relative expression of IGF2BP2 was detected in AML patients by qRT-PCR. (**C**) Relative expression of IGF2BP2 was measured in AML of TCGA database. (**D**) The KM curve was used to predict the effect of IGF2BP2 on the prognosis of AML. (**E**) qRT-PCR was performed to determine expression of IGF2BP2 in FLT3-ITD + or FLT3-ITD- cells. *P < 0.05

To explore specific m6A-relative genes that were differentially expressed between FLT3-ITD + patients and FLT3-ITD-, 11 FLT3-ITD + samples and 101 FLT3-ITD- samples from AML patients were performed to subject to microarray assay analysis. The differently expressed m6A-relative genes were identified to base on the fold change more than 2. Heatmap and box plot were shown that the 23 m6A-relative genes expression in sample tissues (Fig. 1A and Supplementary Fig. 1). We found that the expression of YTHDC1 and IGF2BP2 was increased in FLT3-ITD + samples and the difference was statistically significant. We further selected IGF2BP2 as our subsequent research object through literature and pre-experiment. Firstly, we verified the elevated expression of IGF2BP2 in FLT3-ITD + patients and AML patients in clinical samples (Fig. 1B-C, P < 0.05). In addition, we performed the same verification on the TCGA database, and our control data were obtained from the GTEx database samples. Our results showed that IGF2BP2 expression was significantly elevated in LAML (Fig. 1D, P < 0.05). Furthermore, KM curve analysis showed that high expression of IGF2BP2 was associated with poor prognosis in AML patients (Fig. 1E, HR = 2.9, *P* = 0.03). Finally, compared with FLT3-ITD- THP-1 and U937 cells, the expression of IGF2BP2 was significantly increased in FLT3-ITD + Molm-13 and MV4-11 cells (Fig. 1F, P < 0.05). These evidences indicate that IGF2BP2 was highly expressed in FLT3-ITD + patients and in FLT3-ITD + cell lines (Molm-13, MV4-11).

### Overexpression IGF2BP2 could promoted the proliferation, epithelial-mesenchymal transition (EMT) process and inhibited apoptosis of MV4-11 and Molm-13 cells

**Fig. 2:**
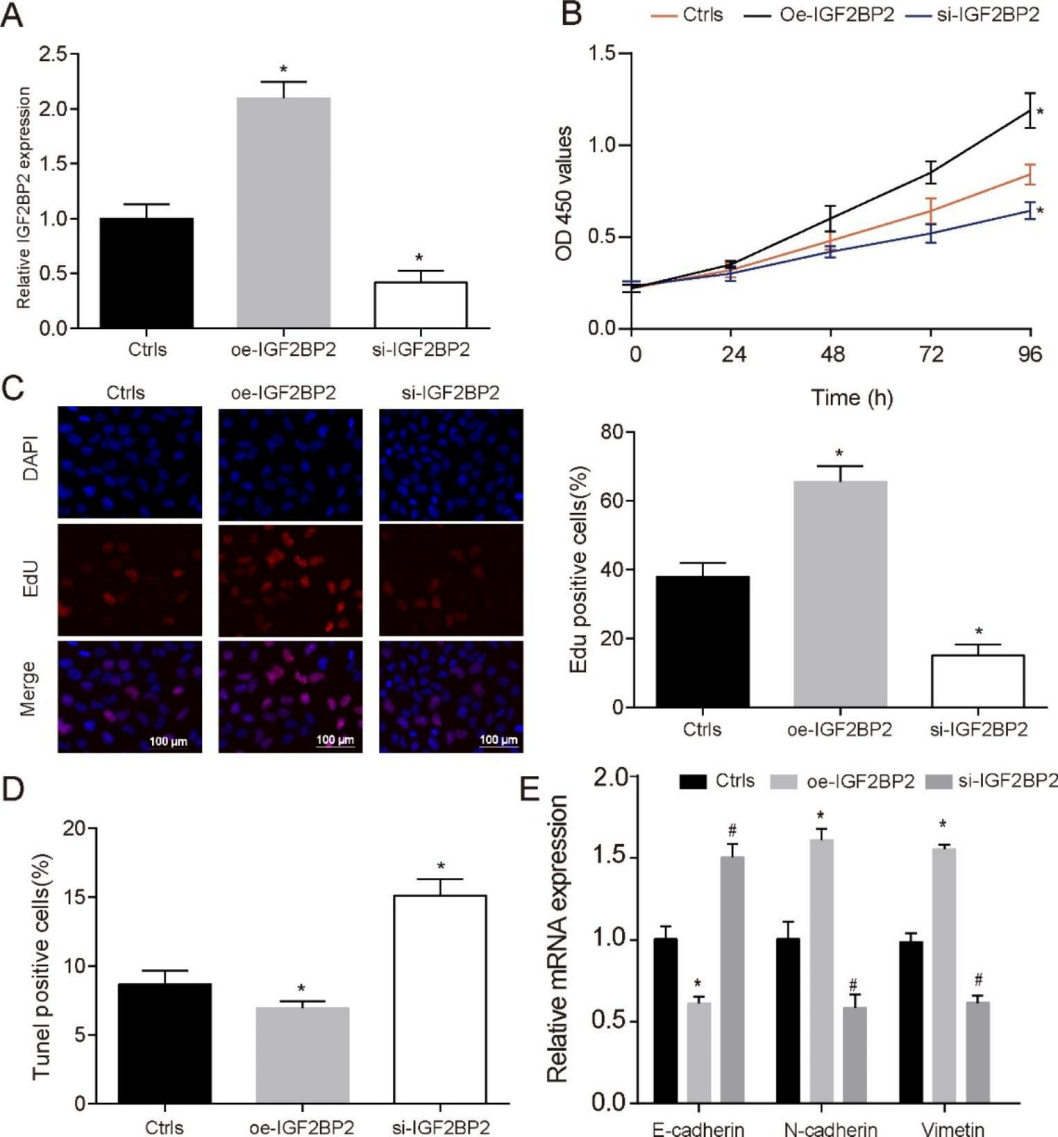
**The influence of different expression levels of IGF2BP2 in MV4-11 cells proliferation and apoptosis** (**A**) qRT-PCR was conducted to determine the transfection efficiency of IGF2BP2. (**B**) MTT was used to detect cell viability in different treatment groups. (**C**) EdU staining was used to test the cell viability in the different groups. Scale bars represent 100 μm. (**D**) Tunel staining was used to test the cell apopotsis in the different groups. Scale bars represent 100 μm. (**E**) Relative expression of EMT biomarkers (E-cadherin, N-cadherin and vimetin) were detected in AML patients by qRT-PCR. *P < 0.05

To further explore the action mechanism of IGF2BP2 expression on FLT3-ITD + AML, we investigated the effects of IGF2BP2 expression on cells proliferation and apoptosis by using MV4-11 and Molm-13 cells. QRT-PCR assay identified that IGF2BP2 expression was significantly up-regulated in MV4-11 cells transfected with oe-IGF2BP2, while its expression was reduced in si-IGF2BP2 group compared with Ctrls group (Fig. 2A, P < 0.05). The results of MTT analysis showed that oe-IGF2BP2 could significantly promote cell proliferation, while inhibiting its expression showed the opposite result. This was also confirmed in Edu staining (Fig. 2B-C, P < 0.05). In addition, apoptosis rate of cells was investigated by Tunel staining, and the result revealed that si-IGF2BP2 was involved in MV4-11 cells apoptosis. Briefly, si-IGF2BP2 elevated apoptosis rate of MV4-11 cell, oe-IGF2BP2 inhibited cells apoptosis (Fig. 2D, P < 0.05). Previous studies have shown that EMT process plays an important role in AML metastasis [19–21]. Therefore, in order to explore the regulation of IGF2BP2 on tumor metastasis, we further examined the expression of EMT markers and found that oe-IGF2BP2 could promote EMT process, while si-IGF2BP2 had the opposite effect (Fig. 2E, P < 0.05). In addition, we performed the same verification in Molm-13 cells. Consistent with our expectations, we see the same trend (Supplementary Fig. 2). This phenomenon suggests that IGF2BP2 can affect the progression of FLT3-ITD + acute myeloid leukemia.

### Glycolysis and the role of PKM in the regulation of MV4-11 cell progression

**Fig. 3:**
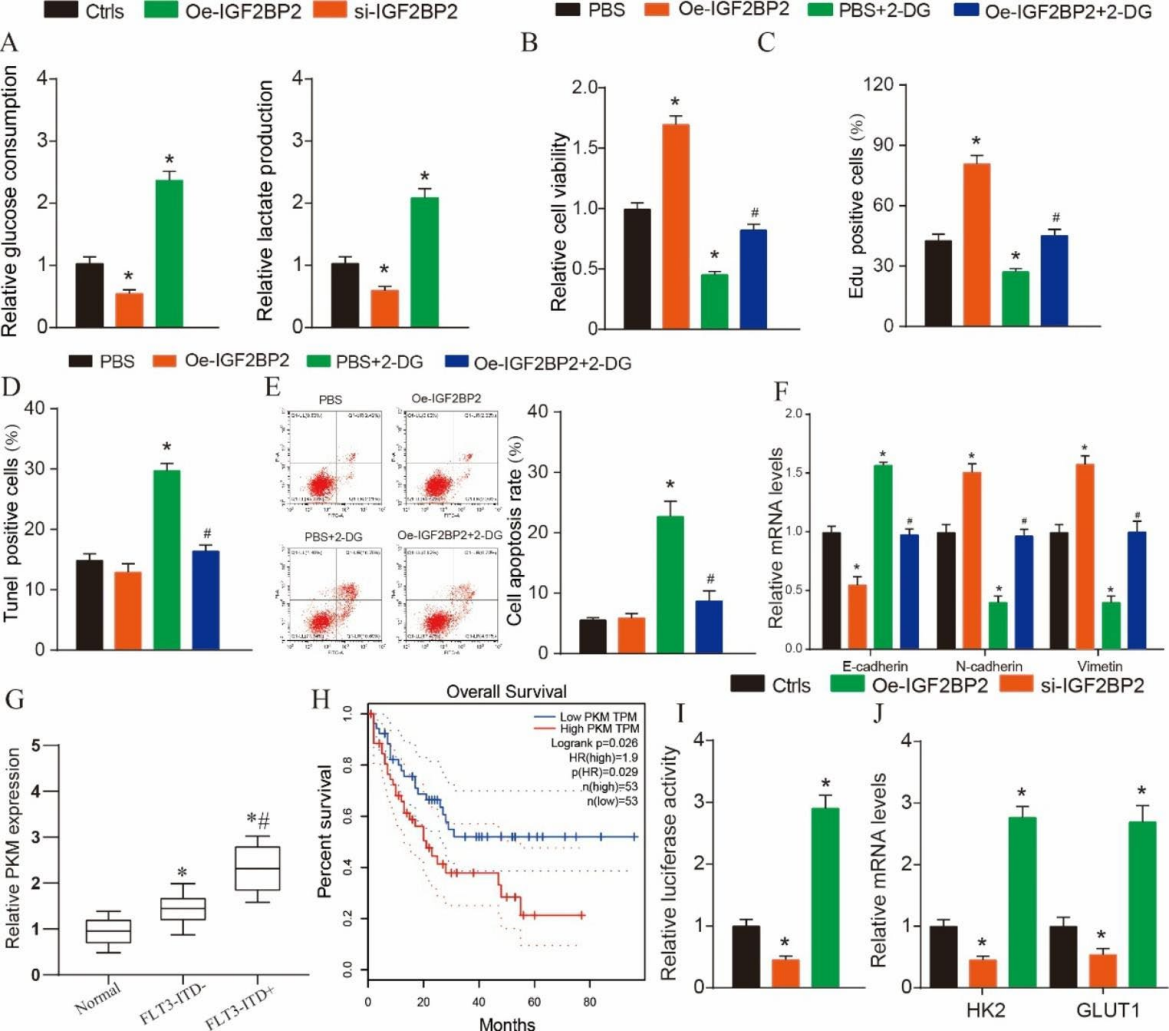
**Glycolysis and the role of PKM in the regulation of MV4-11 cell progression** (**A**) Glucose consumption and lactate production were used to verify the effect of IGF2BP2 on glycolysis. (**B**) MTT was used to detect cell viability in different treatment groups. (**C**) EdU staining was used to test the cell viability in the different groups. Scale bars represent 100 μm. (**D**) Tunel staining was used to test the cell apopotsis in the different groups. Scale bars represent 100 μm. (**E**) Flow cytometry was used to test the cell apopotsis in the different groups. (**F**) Relative expression of EMT biomarkers (E-cadherin, N-cadherin and vimetin) were detected in AML patients by qRT-PCR. (**G**) Relative expression of PKM was detected in AML patients by qRT-PCR. (**H**) The KM curve was used to predict the effect of PKM on the prognosis of AML. (**I**) Double fluorescein was used to verify the binding of IGF2BP2 and HIF-1a. (**J**) Relative expression of HK2 and GLUT1 mRNA were detected in different group by qRT-PCR.*P < 0.05.

Studies had shown that glycolysis plays an important role in AML progression, but its role in FLT3-ITD + AML and its relationship with IGF2BP2 have not been demonstrated [22–24]. Therefore, we further examined glucose consumption and lactate production and found that oe-IGF2BP2 could promote glucose consumption and lactate production (Fig. 3A, P < 0.05). To further verify the role of glycolysis in tumor progression. We selected glycolytic inhibitor 2-DG to treat MV4-11 cells and found that it could reverse the pro-proliferation and anti-apoptosis effects of oe-IGF2BP2 on MV4-11 cells. This is also proved in further EMT process detection (Fig. 3B-F, P < 0.05). These results demonstrated that glycolysis was involved in the regulation of FLT3-ITD + AML progression by IGF2BP2. PKM is known to play an important role in the regulation of tumor glycolysis and is able to activate HIF-1α-dependent transcription of glycolytic enzymes. Further, using clinical samples and TCGA data, we found that PKM expression was increased in tumor tissues, and its high expression was associated with poor prognosis (Supplementary Fig. 3, Fig. 3G-H, P < 0.05). Therefore, our further luciferase assay results showed that IGF2BP2 knockdown could reduce the reporter activity of HIF-1α, and IGF2BP2 overexpression could increase the reporter activity of HIF-1α (Fig. 3I, P < 0.05). Low expression of IGF2BP2 down-regulated HK2 and GLUT1 mRNA expression in MV4-11 cells (Fig. 3J, P < 0.05). Therefore, our data show that IGF2BP2 promotes glycolysis in a PKM/ HIF-1α-dependent manner, thereby promoting FLT3-ITD + AML progression.

### IGF2BP2 affects DANCR RNA stability

**Fig. 4:**
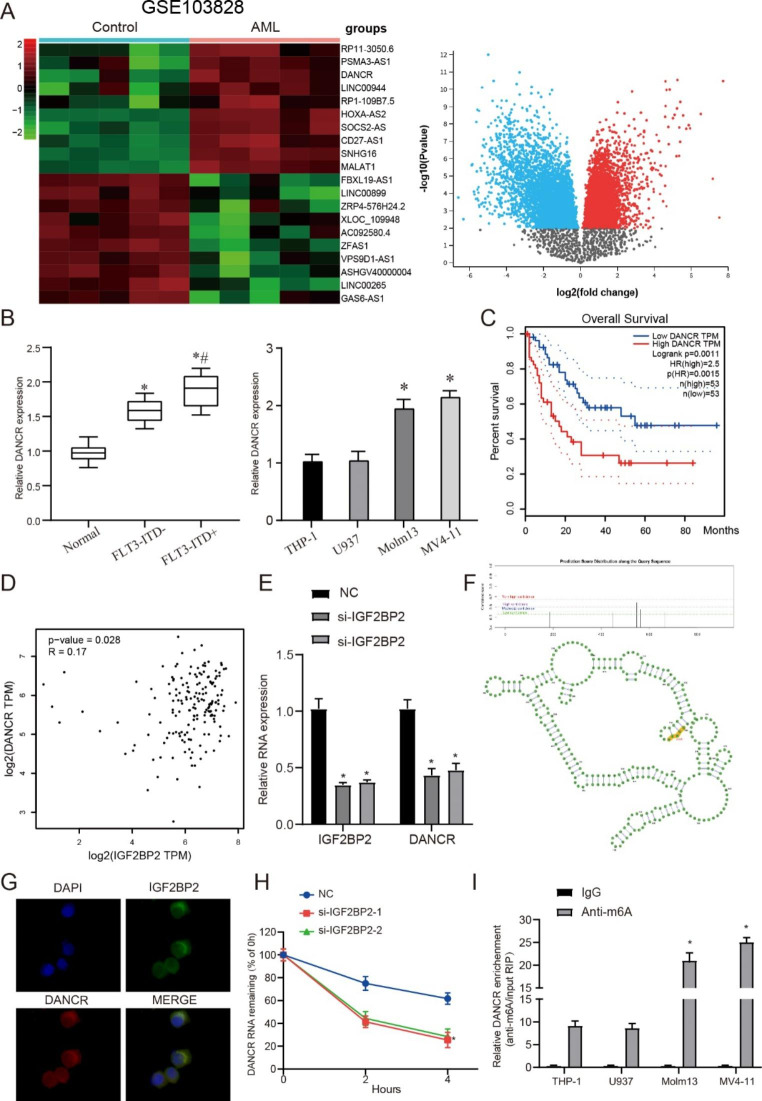
**IGF2BP2 affects DANCR RNA stability** (**A**) The heat map and volcano map showed the top 10 most increased and 10 decreased lncRNAs in AML patients as compared to that in the control samples analyzed by lncRNAs Arraystar Chip. (**B**) Relative expression of DANCR was measured in AML patients and cells. (**C**) Analysis of the prognosis of AML with different expression of DANCR by KM curve. (**D**) Pearson correlation analysis of the correlation between IGF2BP2 and DANCR in clinical samples. (**E**). QRT-PCR detects the expression of IGF2BP2 and DANCR after si-IGF2BP2 treatment. (**F**) SRAMP database was used to verify the m6A binding site and RNA secondary structure on DANCR. (**G**) FISH assay was used to verify the substructural localization of IGF2BP2 and DANCR in MV4-11 cells. (**H**). Compared with the control, the stability of DANCR RNA was reduced in MV4-11 cells with knockout of IGF2BP2 gene. (**I**) The change of m6A-modified DANCR increased when IGF2BP2 interfered with the expression. *P < 0.05

As described in the introduction, a large number of studies have reported that m6A modification is involved in the regulation of ncRNA generation process, affecting RNA splicing and stability, including lncRNA and mRNA. At present, a variety of lncrnas have been confirmed to be elevated due to m6A regulation. Therefore, 5 control samples and 5 AML patient samples from GEO database were used for lncRNA microarray analysis. Our results showed 20 most abnormally expressed lnRNAs, of which 10 were down-regulated and 10 were up-regulated (Fig. 4A). Based on the existing results, we selected DANCR for clinical samples and cell detection and found that its expression was significantly increased in FLT3-ITD + samples (Fig. 4B, P < 0.05). Furthermore, high expression of DANCR was associated with poor prognosis (Fig. 4C, P < 0.05). At the same time, Pearson correlation was used to analyze the expression of IGF2BP2 and DANCR and found that there was a positive correlation (Fig. 4D, P < 0.05). In addition, DANCR expression was significantly down-regulated when IGF2BP2 expression was inhibited (Fig. 4E, P < 0.05). We found that DANCR had multiple m6A binding sites by database analysis (Fig. 4F). Further FISH detection revealed that IGF2BP2 and DANCR were co-expressed in MV4-11 cells and mainly expressed in the cytoplasm (Fig. 4G). The half-life assay also found that DANCR decay rate increased when IGF2BP2 expression was inhibited (Fig. 4H, P < 0.05). Finally, M6A-RIP was used to verify that DANCR had significant m6A methylation (Fig. 4I, P < 0.05). These data suggest that IGF2BP2 is involved in DANCR stability as a reading protein. This may also explain the elevated expression of DANCR in tissues and cells.

### MiR-4701-5p was a regulator of DANCR and PKM

**Fig. 5:**
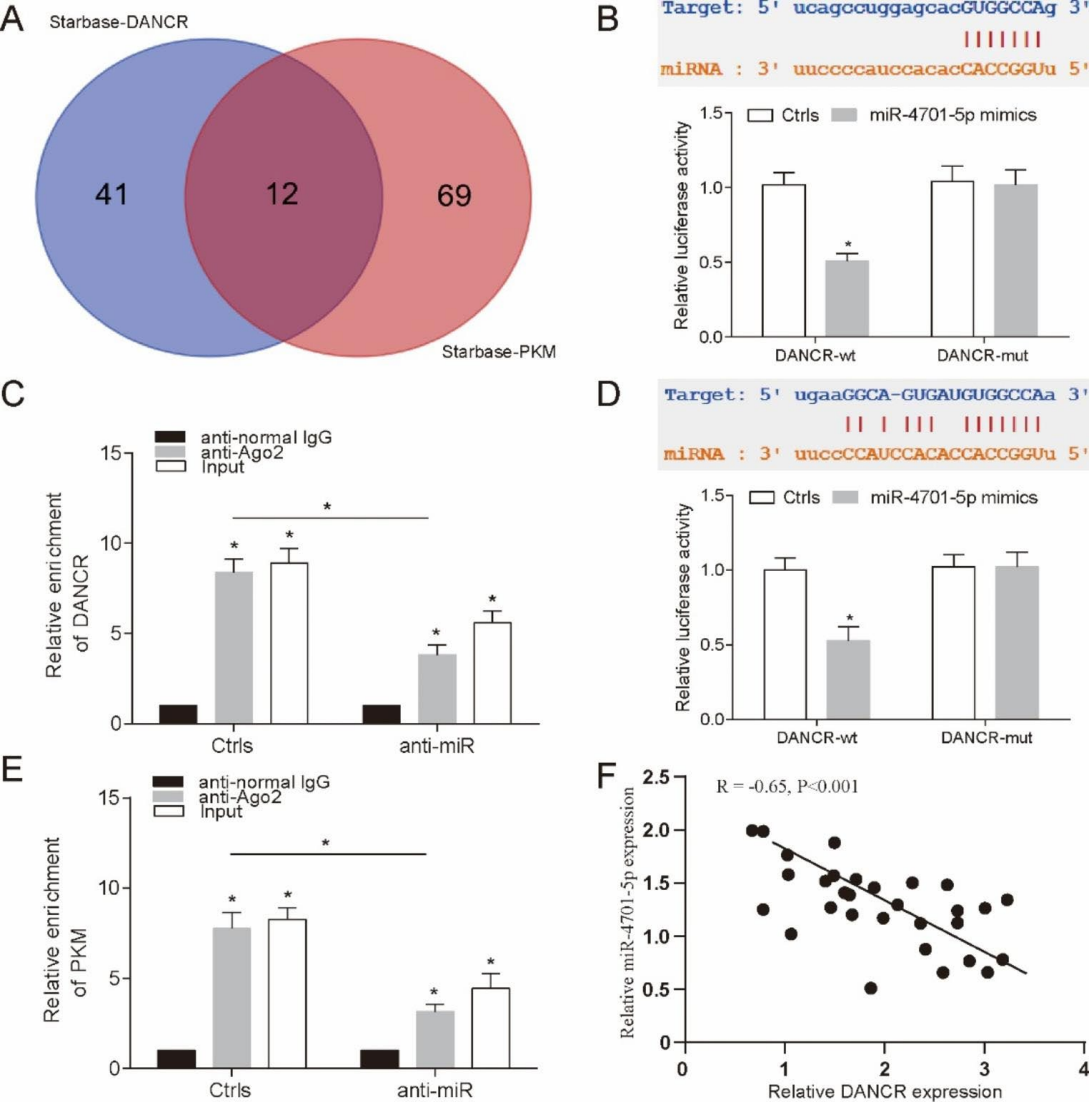
**MiR-4701-5p was a regulator of DANCR and PKM** (**A**) Draw Venn diagram for targeted miRNA screening of DANCR and PKM. (**B**) Dual luciferase assay verified that miR-4701-5p was direct target of DANCR. (**C**) RIP was used to detect the enrichment of DANCR after miR-4701-5p treatment. (**D**) Dual luciferase assay verified that PKM was direct target of miR-4701-5p. (**E**) RIP assay was used to detect the enrichment of PKM after miR-4701-5p treatment. (**F**) Pearson correlation analysis of the correlation between DANCR and miR-4701-5p in FLT3-ITD + AML. *P < 0.05

In order to further explore the relationship between DANCR and PKM and its biological role, this study can also provide a downstream basis for the mechanism exploration of IGF2BP2. Therefore, we tried to verify whether competitive adsorption mechanism (ceRNA) mechanism plays an important role in it. Firstly, we used Starbase database to enrich and analyze the targeted miRNAs of DANCR and PKM and found 12 kinds of co-targeted mirnas. Further, we verified the expression of these twelve miRNAs by pre-experiment and found that miR-4701-5p may play a regulatory role in them (Fig. 5A). Both dual-luciferase reporter gene detection and RIP experiments confirmed our conjecture, and the results showed that DANCR had a targeting relationship with miR-4702-5p (Fig. 5B-C, P < 0.05). This was also confirmed in the targeting of miR-4701-5p and PKM (Fig. 5D-E, P < 0.05). This suggests that miR-4701-5p may be an intermediate regulator of DANCR and PKM. Finally, we found a negative correlation between the expression of DANCR and miR-4701-5p in FLT3-ITD + AML patients, which further verified our conjecture (Fig. 5F, P < 0.05).

**DANCR affects the proliferation and glycolysis of MV4-11 cells by regulating the expression of PKM through miR-4701-5p**.

**Fig. 6:**
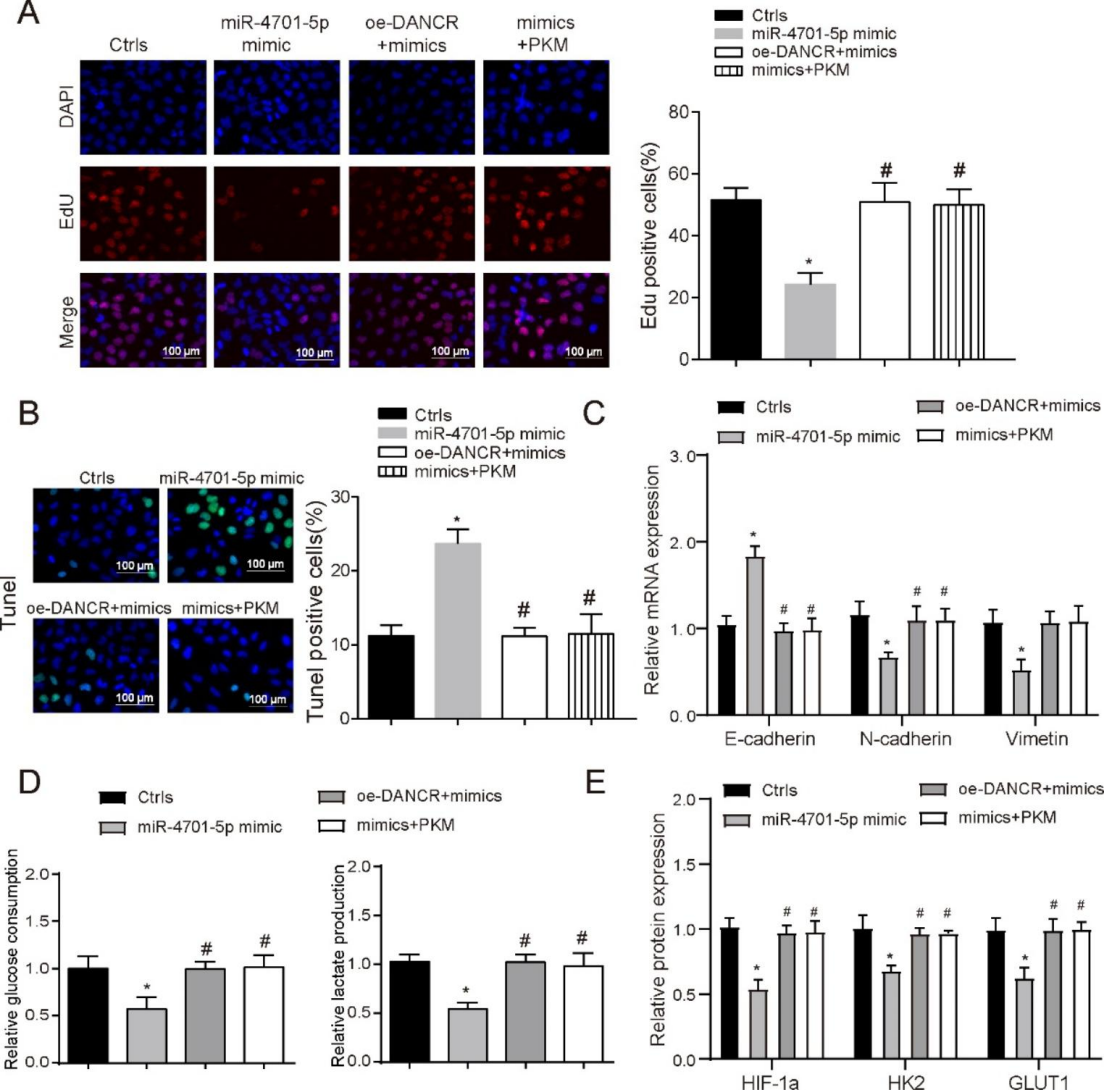
**DANCR affects the proliferation and glycolysis of MV4-11 cells by regulating the expression of PKM through miR-4701-5p** (**A**) EdU staining was used to test the cell viability in the different groups. Scale bars represent 100 μm. (**B**) The effect of DANCR, miR-4701-5p and PKM on cells apoptosis was investigated by using TUNEL staining. (**C**) Relative expression of EMT biomarkers (E-cadherin, N-cadherin and vimetin) were detected in different group by qRT-PCR. (**D**) Glucose consumption and lactate production were used to verify the effect of different treatmented on glycolysis. (**E**) WB was used to detect the protein expression of HIF-1a, HK2 and GLUT1 in each treatment group. *P < 0.05

To determine the modulated capacity of miR-4701-5p in progression of FLT3-ITD + AML, this research explored the effects of miR-4701-5p on proliferation and apoptosis of MV4-11 cells. MV4-11 cells were co-transfected with Lipofectamine 2000 (Ctrls), miR-4701-5p mimics, oe-DANCR + miR-4701-5p-mimics or PKM + miR-4701-5p-mimics (Supplementary Fig. 4). As shown in Fig. 6A, Edu staining assay found that Edu positive cell rate decreased in miR-4701-5p mimics group and the effect was reversed by DANCR or PKM (*P* < 0.05). Cells apoptosis were detected by using Tunel staining. Results showed that miR-4701-5p significantly promoted apoptosis ability of MV4-11 cells (Fig. 6B, P < 0.05). This effect also applies to the detection of glycolysis levels and EMT progress (Fig. 5C-D, P < 0.05). Finally, WB was used to verify that the HIF-1 A/GLUT1 pathway was also regulated by DANCR/miR-4701-5p/PKM (Fig. 5e, P < 0.05). These data suggest that DANCR regulates MV4-11 progression via miR-4701-5p/PKM.

***I in vivo*assays to verify the regulatory effect of IGF2BP2 on FLT3-ITD + AML progression**.

**Fig. 7:**
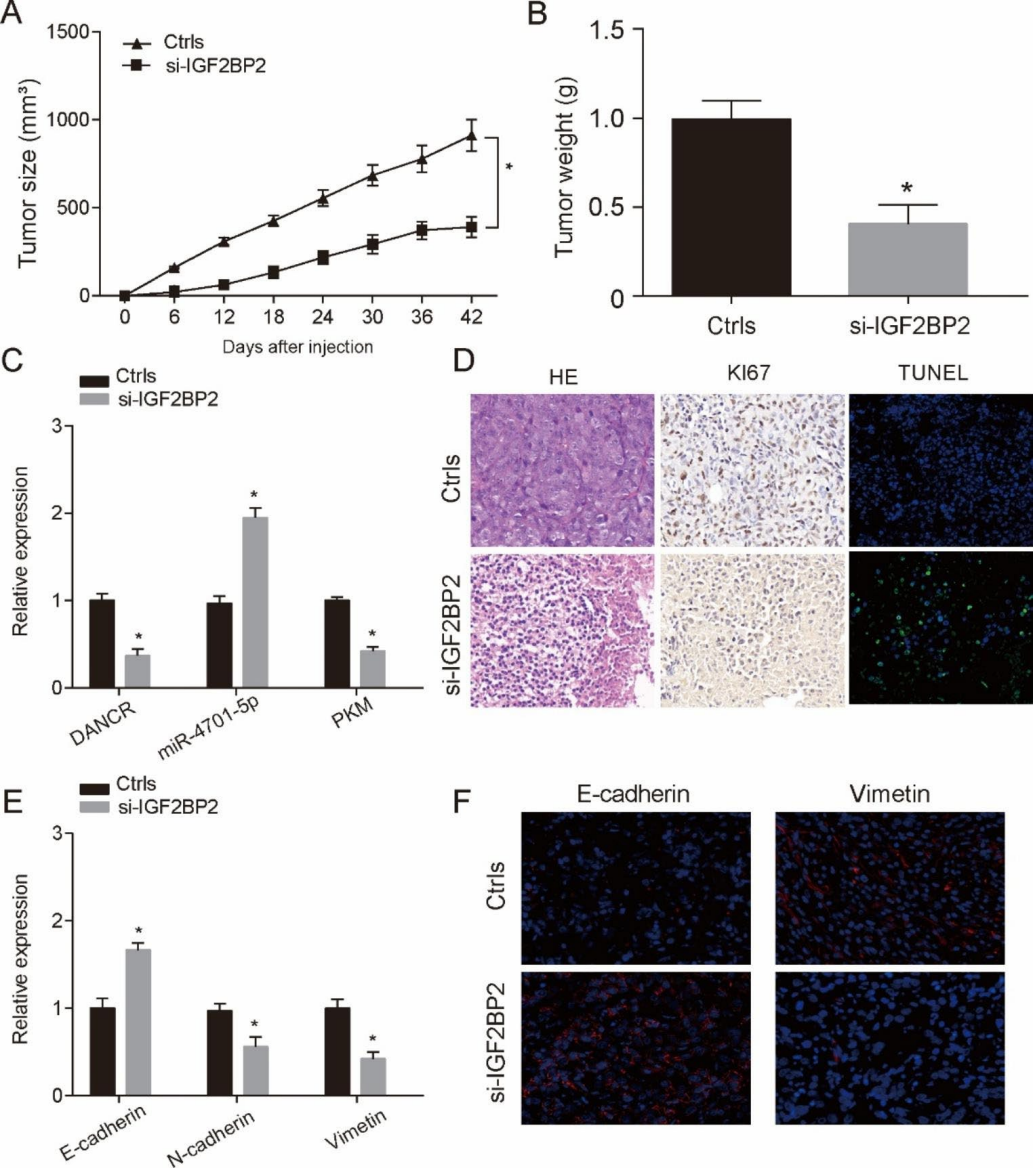
In vivo**assays to verify the regulatory effect of IGF2BP2 on FLT3-ITD + AML progression** (**A**) The tumor size was measuserd every 6 days for 42 days, and tumor volume was calculated. (**B**) Tumors were excided from nude mice who were injected with MV4-11 cells transfected with ctrl or si-IGF2BP2. The tumor weight were measured. (**C**) QRT-PCR assay was used to examine expreesions of DANCR, miR-4701-5p and PKM for different transfected groups in vivo. (**D**) Expression of Ki67 and TUNEL in each group was detected by immunohistochemical staining. (**E**) Relative expression of EMT biomarkers (E-cadherin, N-cadherin and vimetin) were detected in different group by qRT-PCR. (**F**) Expression of E-cadherin and Vimetin in each group was detected by immunohistochemical staining. *P < 0.05

From the view of growth curve of tumor size, we apparently found that knockdown IGF2BP2 inhibited tumors growth compared with Ctrls group (Fig. 7A, P < 0.05). Tumors were weighted after excised, and we observed that tumors’ weight in si-IGF2BP2 group was evidently lower than that in Ctrls group (Fig. 7B, P < 0.05). Afterwards, qRT-PCR was performed to quantitate the expression level of DANCR, miR-4701-5p and PKM, as shown in Fig. 7C. The results showed that si-IGF2BP2 inhibited the expression of DANCR and PKM and promoted the expression of miR-4701-5p. We performed morphological examination on the collected tumor samples and found that si-IGF2BP2 inhibited proliferation and promoted apoptosis in tumor tissue (Fig. 7D). Finally, in order to verify the EMT process in vivo, we used qRT-PCR and immunofluorescence to verify the expression of EMT markers and found that inhibition of IGF2BP2 could inhibit the metastasis process of tumors (Fig. 7E-F). These data suggest that IGF2BP2 could regulate disease progression by affecting DANCR stability.

## Discussion

AML is a malignant clonal disease in which hematopoietic stem/progenitor cells accumulate various acquired genetic aberrations, leading to changes in blood cell growth/differentiation [25]. Due to the complexity and heterogeneity of the pathogenesis of AML, it still cannot be fully reflected in the classification system at present. The pathogenesis, classification, diagnosis and treatment of AML are facing a research bottleneck. In recent years, the establishment of “epigenetic transcriptomics” has provided a new research direction for the diagnosis and treatment of AML [26]. “Epigenetic transcriptomics” is a new way of regulating gene expression found at RNA level. N6-methyladenosine (m6A) modification is the most common post-transcriptional modification of eukaryotic mRNAs and lncRNAs [9, 27]. Since the first discovery of m6A deposition in AML and its direct involvement in carcinogenesis. The reversibility and dynamic nature of m6A modification, as well as its ability to fine-tune and coordinate gene expression programs, have greatly contributed to leukemic cell differentiation, development, and clinical diagnosis and treatment of AML [8, 28]. In this context, this study first found that IGF2BP2, a reading protein in m6A, is highly expressed in FLT3-ITD + AML patients and cells, and its regulatory role in tumor progression and glycolysis. Further study of the mechanism revealed that IGF2BP2 could regulate glycolytic level by stabilizing the expression of DANCR and mediate miR-4701-5p/PKM pathway, thereby affecting the progression of FLT3-ITD + AML.

At present, it has been found that IGF2BP2 expression is increased in AML, and a large number of studies have found that IGF2BP2 can be involved in regulating the stability of lncRNA and affecting its expression [14, 29, 30]. We analyzed the m6A-related genes of FLT3-ITD + patients and FLT3-ITD- patients by TCGA database data, and found that IGF2BP2 expression was up-regulated, and the difference was statistically significant. In this study, we demonstrated for the first time the role of IGF2BP2 in AML and glycolysis. LncRNA usually plays an important regulatory role in many biological processes of tumor cells, such as proliferation, invasion, migration, apoptosis, senescence and drug resistance [31–34]. Differentiation antagonistic non-protein-coding RNA(DANCR) is a common lncRNA in the cytoplasm, which exists on human chromosome 4q12. At present, previous studies have shown that DANCR expression is significantly increased in a variety of tumors and has cancer-promoting effects [17, 18]. Other studies have shown that DANCR activates autophagy through the miR-874-3P/ATG16L1 axis, resulting in cytabine resistance in AML. However, no studies have reported the role of DANCR in FLT3-ITD + AML [35, 36]. At present, studies have demonstrated the role of m6A methylation in promoting lncRNA stability. We used RNA half-life assay and m6A-RIP assay to verify that IGF2BP2 could stabilize and promote the expression of DANCR. This also partly explains the elevated expression of DANCR.

In the process of tumorigenesis, the energy metabolism of tumor cells is abnormal, which is characterized by overactive glycolysis and weakened mitochondrial aerobic metabolism. The whole metabolic network is reprogrammed under the dominance of tumor genes and tumor suppressor genes. Just as the “Warburg effect” proposed by Warburg in 1920 pointed out that tumor cells need to undergo metabolic reprogramming to meet their own rapid proliferation needs: when oxygen is sufficient, tumor cells prefer to adopt glycolysis to metabolize glucose instead of mitochondrial oxidative phosphorylation, which can produce more ATP [37]. Warburg effect is commonly found in various malignant tumor cells and tumor tissues, such as liver cancer, gastric cancer, breast cancer, leukemia cells, colon cancer cells and brain malignant tumors [37–39]. Inhibition of glycolysis can inhibit the proliferation of tumor cells and kill tumor cells, so as to achieve the goal of anti-tumor. It has been found that the activity of glycolysis in malignant tumors is closely related to the enzymes that catalyze glycolysis. The expression and activity of key glycolytic enzymes, such as hexokinase-2 (HK2), glucose transporter type 1 (GLUT1) and pyruvatekinase M (PKM), can affect the glycolysis of tumor cells and thereby affect tumor proliferation [40, 41]. Our study found that IGF2BP2 could regulate glycolytic levels in MV4-11 cells. Further examination revealed that PKM functions through the HIF-1a/GLUT1 pathway. This role again provides a mechanistic explanation for the biological function of IGF2BP2 in FLT3-ITD + AML.

Current studies have found that competitive adsorption mechanism lncRNAs are involved in an important process of tumor progression regulation [42]. Further, our results showed that DANCR was mainly expressed in the cytoplasm, which further confirmed our conjecture. Therefore, we sought to investigate the role of miRNAs in two-molecule tandem regulation. The miRNA profiles between PKM and DANCR were predicted by Starbase database. Finally, through the preliminary experimental data, we found that miR-4701-5p may be their potential regulatory mediator. This conjecture was confirmed by the subsequent double luciferase reporter and RIP experiments. By searching the available data, we found for the first time that miR-4701-5p may be involved in the regulation of glycolysis and disease progression in FLT3-ITD + AML as a target gene of DANCR. This data will support early disease screening and targeted therapies in the future. However, although our data have confirmed our conjecture at both the in vivo and in vitro levels, we still lack the necessary clinical cohort data to confirm the data including the biomarker and target regulation conjecture in the population. Although this is something that we are currently preparing for and it will be one of our main research directions in the future.

## Conclusion

In short, our study was the first found the IGF2BP2 could promotes the progression of FLT3-ITD + AML by regulating the expression of DANCR and regulating the glycolysis level of miR-4701-5p/PKM pathway. This discovery will provide new horizons for early screening and targeted therapy of FLT3-ITD + AML.

## Electronic supplementary material

Below is the link to the electronic supplementary material.

Supplementary Material 1

Supplementary Material 2

Supplementary Material 3

## Data Availability

The datasets used and/or analysed during the current study available from the corresponding author on reasonable request.
