# Supplementary material for: IGF2BP2 promotes lncRNA DANCR stability mediated glycolysis and affects the progression of FLT3-ITD + acute myeloid leukemia
Source: Apoptosis. 2023 Apr 15;28(7-8):1035–47. doi: 10.1007/s10495-023-01846-0 (PMC10333402; doi:10.1007/s10495-023-01846-0)
Supplement: Supplementary file 1 — Supplementary Material 1 [file 10495_2023_1846_MOESM1_ESM.docx]

**Expression profile analysis of lncRNAs**

The lncRNA chip analysis data came from the GEO database (GSE103828). The 10 samples got analyzed using lncRNAs chips. In the study presented here, 5 pairs of AML patients and iron deficiency anemia (IDA) controls were screening by microarray. Labeled RNAs were scanned and acquired by employing an Agilent-079487 Arraystar Human LncRNA microarray V4 (Agilent Technologies, USA), and array images were analyzed by Agilent Feature Extraction software (version 11.0.1.1, USA). The differentially expressed lncRNAs were selected according to the fold-change cut-off (fold change≥2) and *P*-value <0.05.

**The Cancer Genome Atlas (TCGA) database analysis**

RNAseq data (level 3) of 112 AML samples (11 FLT3-ITD+ patients and 101 FLT-ITD wild type patients) were obtained from The Cancer Genome Atlas (TCGA) dataset (https://portal.gdc.com) and corresponding clinical information. m6A-related genes were derived from Juan Xu et al.'s study on the molecular characterization and clinical significance of m6A regulators across 33 cancer types.

The above results were achieved with the R (v4.0.3) packages ggplot2 and pheatmap.

**Edu Staining**

To measure proliferation potential of cells, cells were subjected to the Cell-Light TM EdU imaging detecting kit (RiboBio, Shanghai, China). Briefly, each well of a 96-well plate contained cells (1×10^5^/well). After being cultured with 10 mM EdU for 2 h, all the Cells were fixed with 4% paraformaldehyde, followed by permeabilization with 0.2% Triton X-100 and stained by Apollo solution for 30 min in the dark. Subsequently, cell nuclei got stained by DAPI solution.

**Dual luciferase activity assay**

Subcloned into luciferase reporter psiCHECK2 (Promega, Madison, WI). On 96-well plates, cells were seeded in the density of 3×10^4^ cells per well for 24 h in triplicate before transfection and then transfected with wild-type or mutated reporter vectors, and miRNA mimics or negative control. Lysates were harvested after being transfected for 24 h.

**RNA Stability Assays**

MV4-11 cells were treated with actinomycin D at a final concentration of 5 μg/ml, and the experiment was divided into control group and IGF2BP2 knockout group. After 0, 2 and 4 hours of treatment, cells in each group were collected for RNA isolation. Then, the expression of DANCR in each treatment group was detected by qRT-PCR.

**RIP assay**

RIP was employed by using a Magna RNA-binding protein immunoprecipitation kit (Millipore, Billerica, MA, USA) on the basis of the manufacturer's instructions. MV4-11 cell lysates containing DANCR, PKM and miR-4701-5p got prepared and incubated with anti-argonaute2 (Ago2) antibody (Millipore). NC was set with Normal mouse IgG (Millipore).

**Immunohistochemistry (IHC)**

Longitudinal sections of tumor specimens were fixed in 10% formalin, embedded in paraffin, and then completed by deparaffinizing with xylene and hydrating with an ethanol gradient. After being successively incubated with antigen retrieval solution (Shunbai, China) and 3% H_2_O_2_ for 30 min, the slides were washed with water and cultured with the primary antibody anti-ki67 (1：100) and anti-TUNEL (Sevier, China) overnight at 4°C. Nonimmunized serum was utilized to dispose negative controls with discarding primary antibody. The next day, secondary antibody (Beijing Biosynthesis Biotechnology Co. Ltd.; Beijing, China) was used to rinse and incubate slides, which then were followed by 3, 3′-diaminobenzidine (DAB) and hematoxylin staining, respectively. Image-J software was performed to analyze the results of DAB staining.
