# Supplementary material for: IGF2BP2 promotes lncRNA DANCR stability mediated glycolysis and affects the progression of FLT3-ITD + acute myeloid leukemia
Source: Apoptosis. 2023 Apr 15;28(7-8):1035–47. doi: 10.1007/s10495-023-01846-0 (PMC10333402; doi:10.1007/s10495-023-01846-0)
Supplement: Supplementary file 2 — Supplementary Material 2 [file 10495_2023_1846_MOESM2_ESM.docx]

Table S1 Primer Sequences for qRT-PCR

| **Genes** | **Sequences（5’-3’）** |
| --- | --- |
| **DANCR** |  |
| Forward primer | GCCACAGGAGCTAGAGCAGT |
| Reverse primer | GCAGAGTATTCAGGGTAAGGGT |
| **miR-4701-5p** |  |
| Forward primer | ATGAGTTGGCCACCACACCTA |
| Reverse primer | ATGAGTTGGCCACCACACCTA |
| **PKM** |  |
| Forward primer | ACAGCCAAAGGGGACTATCCT |
| Reverse primer | AGGACGATTATGGCCCCACT |
| **IGF2BP2** |  |
| Forward primer | 5’- TGAGCCAAAAATTCAGAATACAAGGA -3’ |
| Reverse primer | 5’- TGAGCCAAAAATTCAGAATACAAGGA -3’ |
| **HK2** |  |
| Forward primer | 5’- TGAGCCAAAAATTCAGAATACAAGGA -3’ |
| Reverse primer | 5’- CCAGGCATTCGGCAATGTG -3’ |
| **GLUT1** |  |
| Forward primer | 5’- TGTGGGCATGTGCTTCCAGTA -3’ |
| Reverse primer | 5’- CGGCCTTTAGTCTCAGGAACTTTG -3’ |
| **E-cadherin** |  |
| Forward primer | 5’- ATTTTTCCCTCGACACCCGAT -3’ |
| Reverse primer | 5’- TCCCAGGCGTAGACCAAGA -3’ |
| **N-cadherin** |  |
| Forward primer | 5’-GAGAGGAAGACCA-GGACTATGA-3’ |
| Reverse primer | 5’- CAGTCATCACCACCACCATAC -3’ |
| **Vimetin** |  |
| Forward primer | 5’- AGTCCACTGAGTACCGGAGAC -3’ |
| Reverse primer | 5’- CATTTCACGCATCTGGCGTTC -3’ |
| **GAPDH** |  |
| Forward primer | CTGGGCTACACTGAGCACC |
| Reverse primer | AGTGGTCGTTGAGGGCAATG |
| **U6** |  |
| Forward primer | CTCGCTTCGGCAGCACA |
| Reverse primer | AACGCTTCACGAATTTGCGT |
