# Supplementary material for: IGF2BP2 promotes lncRNA DANCR stability mediated glycolysis and affects the progression of FLT3-ITD + acute myeloid leukemia
Source: Apoptosis. 2023 Apr 15;28(7-8):1035–47. doi: 10.1007/s10495-023-01846-0 (PMC10333402; doi:10.1007/s10495-023-01846-0)
Supplement: Supplementary file 3 — Supplementary Material 3 [file 10495_2023_1846_MOESM3_ESM.docx]

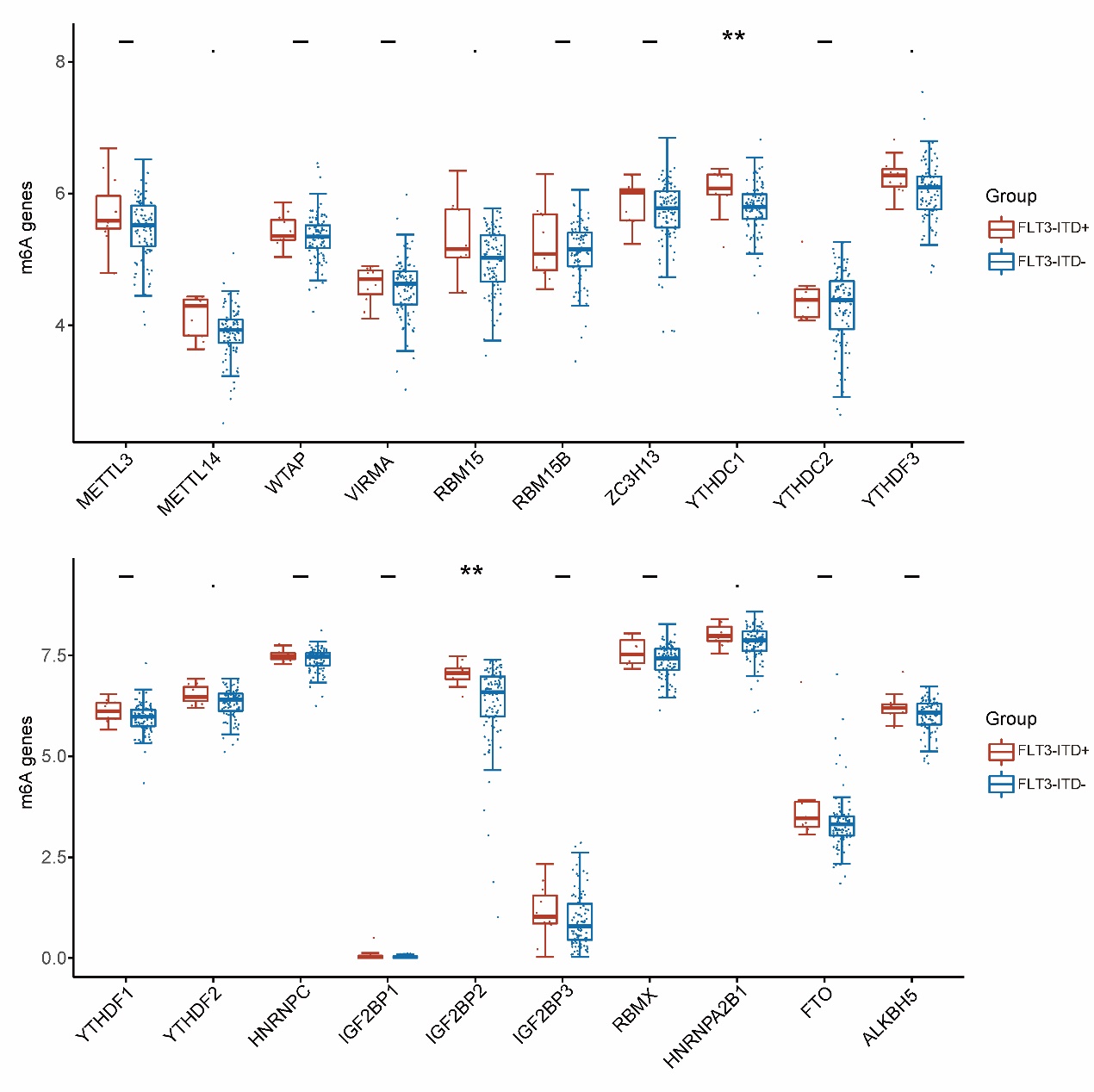


**Supplementary Figure 1 TCGA database analysis of the expression of 23 m6A-related genes in AML before and after treatment.**


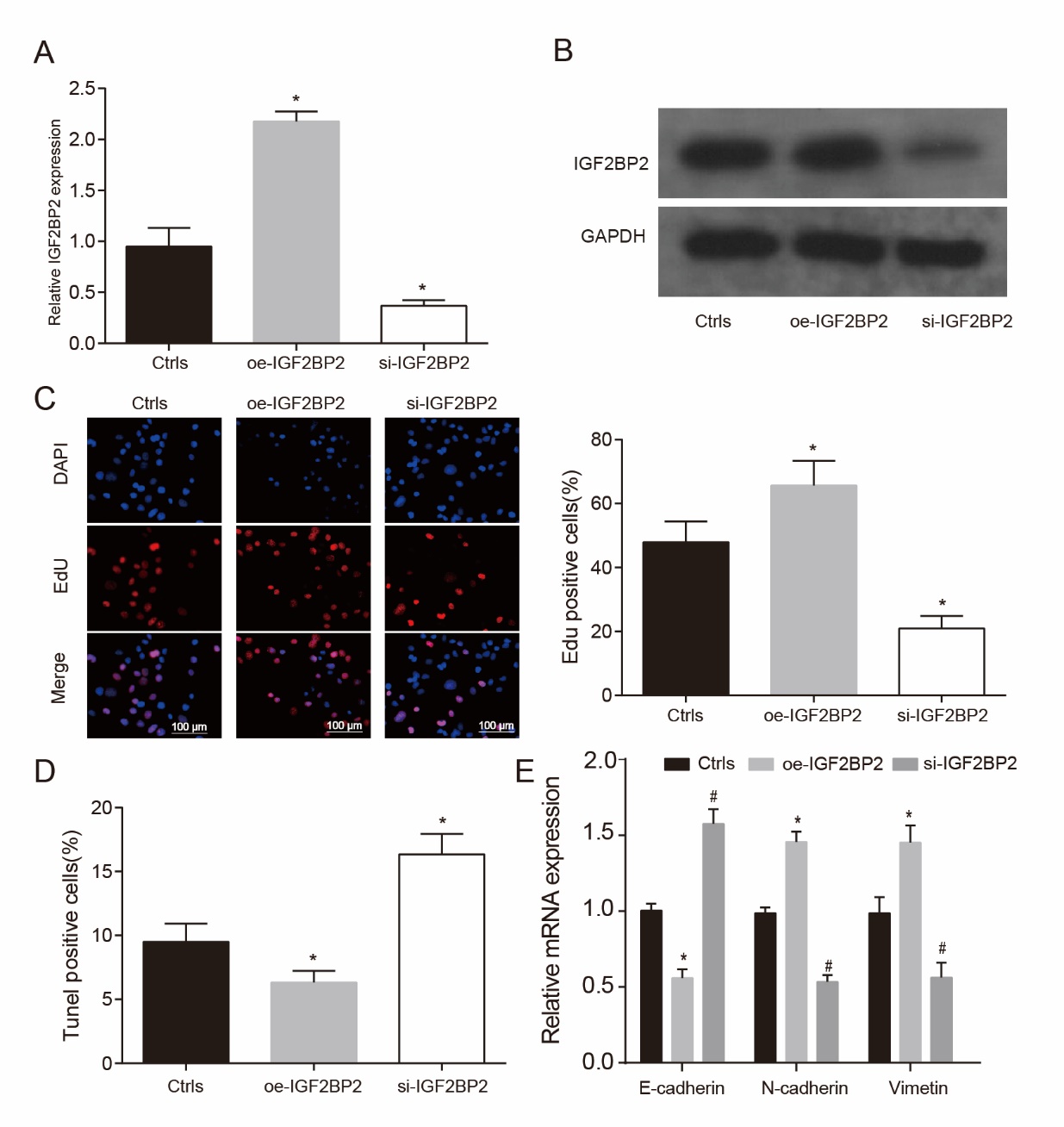


**Supplementary Figure2.** **The influence of different expression levels of IGF2BP2 in Molm-13 cells proliferation and apoptosis**

(A-B) qRT-PCR(A) and WB(B) were conducted to determine the transfection efficiency of IGF2BP2. (C) EdU staining was used to test the cell viability in the different groups. Scale bars represent 100μm. (D) Tunel staining was used to test the cell apopotsis in the different groups. Scale bars represent 100μm. (E) Relative expression of EMT biomarkers (E-cadherin, N-cadherin and vimetin) were detected in AML patients by qRT-PCR. **P*<0.05.


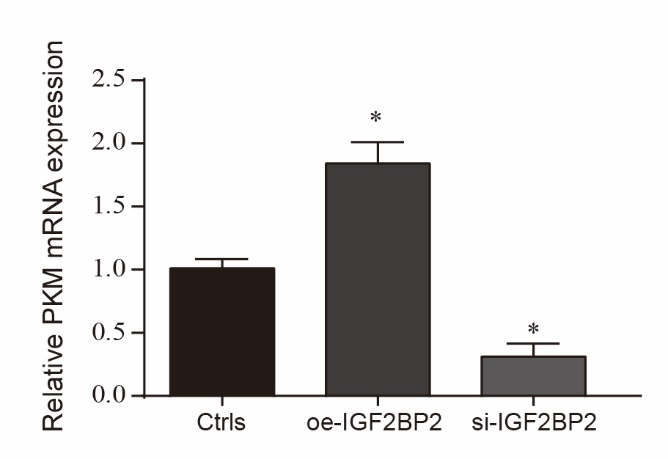


**Supplementary Figure 3 The expressions of PKM were detected by QRT-PCR.**


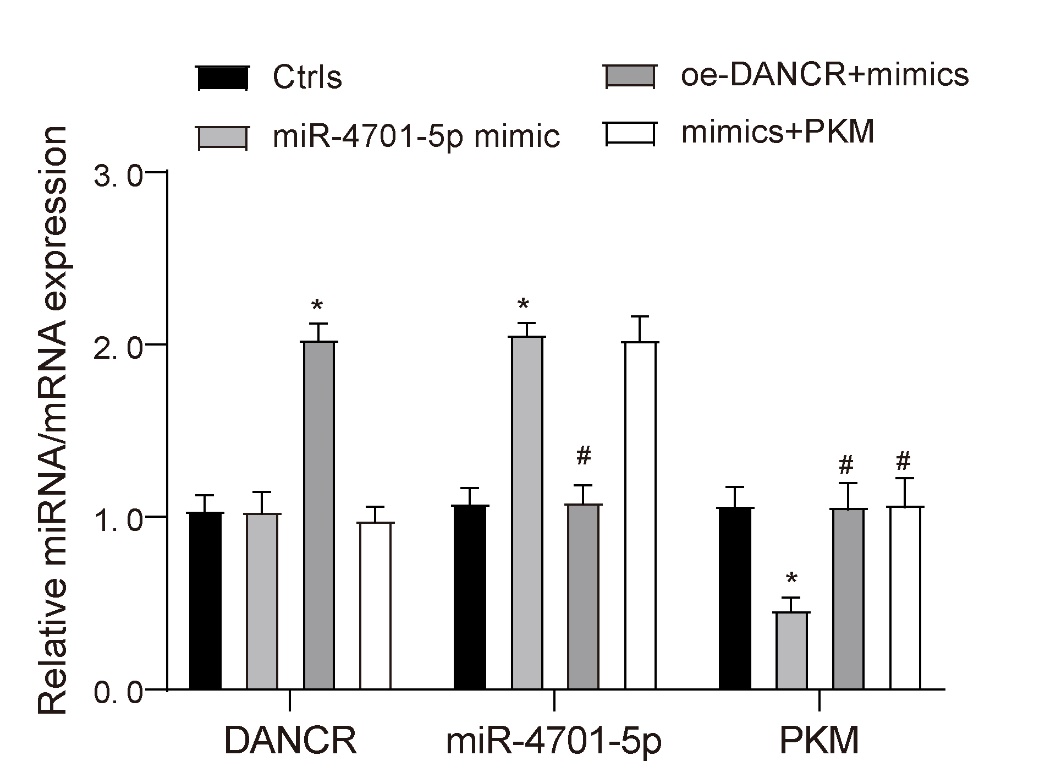


**Supplementary Figure 4 The expressions of DANCR, miR-4701-5p and PKM were detected by QRT-PCR.**
